# Child malnutrition in Haiti: progress despite disasters

**DOI:** 10.9745/GHSP-D-13-00069

**Published:** 2013-11-14

**Authors:** Mohamed Ag Ayoya, Rebecca Heidkamp, Ismael Ngnie–Teta, Joseline Marhone Pierre, Rebecca J Stoltzfus

**Affiliations:** a UNICEF Country Office, Nutrition Section, Port-au-Prince, Haiti; b Johns Hopkins Bloomberg School of Public Health, Department of International Health, Baltimore, MD, USA; c Haiti Ministry of Public Health and Population, National Food and Nutrition Program Coordination Unit, Port-au-Prince, Haiti; d Cornell University, Division of Nutritional Sciences, Ithaca, NY, USA

## Abstract

Undernutrition, a chief child killer in developing countries, has been a major public health problem in Haiti. Following the 2010 disasters (earthquake and cholera) and the intensive relief efforts to address them, we sought to determine the trends of child undernutrition in Haiti using data from the 2005–06 Haiti Demographic and Health Survey (HDHS) and from a Standardized Monitoring and Assessment of Relief and Transitions (SMART) survey in 2012. Growth data analyses included 2,463 (HDHS) and 4,727 (SMART) children ages 0–59 months. We calculated the prevalence of stunting, wasting, and underweight for each survey using World Health Organization 2006 growth standards. To account for sampling design, probability weights were applied to all analyses. Statistical significance was determined by non-overlapping confidence intervals around estimates. Stunting prevalence declined from 28.5% (95% confidence interval [CI] = 25.9, 31.3) in 2005-06 to 22.2% (95% CI = 20.2, 24.3) in 2012; wasting, from 10.1% (95% CI = 8.2, 12.7) to 4.3% (95% CI = 3.6, 5.2); and underweight, from 17.7 % (95% CI = 15.6, 20.1) to 10.5% (95% CI = 9.3, 11.9). Additionally, stunting declined more in rural areas, from 33.6% (95% CI = 30.1, 37.2) in 2005–06 to 25% (95% CI = 23.4, 26.7) in 2012, than in urban areas, from 18.6% (95% CI = 15.3, 22.5) in 2005–06 to 18.4% (95% CI = 16.7, 20.1) in 2012, for reasons that remain unknown. Results of the 2012 HDHS confirmed the observed trends. Thus, undernutrition among Haitian children under 5 declined significantly between 2005–06 and 2012. Our results should be interpreted in view of investments and changes that occurred in different sectors (within and outside health and nutrition) before and after the earthquake.

## Abstract (precis)

Despite a devastating earthquake and a major cholera outbreak in Haiti in 2010, surveys in 2006 and 2012 document marked reductions in child undernutrition. Intensive relief efforts in nutrition as well as synergies and improvements in various sectors before and after the earthquake were likely contributing factors.

## INTRODUCTION

Globally, an estimated 165 million children under age 5 are stunted, and at least 52 million are wasted.^1^ Undernutrition accounts for 45% of all deaths among children under 5 years of age.^1^ Haiti has the highest rates of childhood underweight and wasting in the Latin America and Caribbean region.^2^ The Global Burden of Disease Study 2010 highlighted Haiti also for its high burden of disease and injury and high mortality and for having the world's lowest healthy life expectancy.^3^

Child undernutrition has long been a major public health problem and silent emergency in Haiti. The fourth national Haiti Demographic and Health Survey (HDHS 4), conducted in 2005–06, found that 1 in every 3 children under age 5 was stunted, 1 in every 10 was wasted, and 2 in every 10 were underweight. Stunting rates in this age group were almost twice as high in rural areas as in urban areas.^4^ The latest HDHS, in 2012, reported lower rates of undernutrition among under-5s: 21.9% stunted, 5.1% wasted, and 11.4% underweight.^5^

### The 2010 Earthquake: The Trigger of a New Era for Nutrition in Haiti

The January 2010 earthquake in Haiti caused unprecedented loss in human life and physical infrastructure. It also displaced at least 1.5 million people, putting more children at high risk of undernutrition.^6^ Throughout 2010, intensive emergency response efforts focused on saving children's lives and preventing undernutrition in earthquake-affected areas: Gonaives, Jacmel, Leogane, Petit Goave, and Port-au-Prince.

At the end of 2010, the Haitian government and its partners began intensified efforts to scale up preventive and recuperative community food and nutrition activities and to increase investments in water-sanitation-hygiene (WASH) and immunization in all of the country's 10 departments. As of May 2012, services continued throughout the country in 285 outpatient programs and 16 inpatient stabilization units for children with severe acute malnutrition, 174 baby tents for the promotion of optimal infant feeding practices and counseling for pregnant and lactating women, and 350 supplementary nutrition programs for children with moderate acute malnutrition (see Map for coverage of nutrition services).^7^

The Haitian government and its partners scaled up efforts to improve community food and nutrition after the 2010 earthquake.

**Figure:**
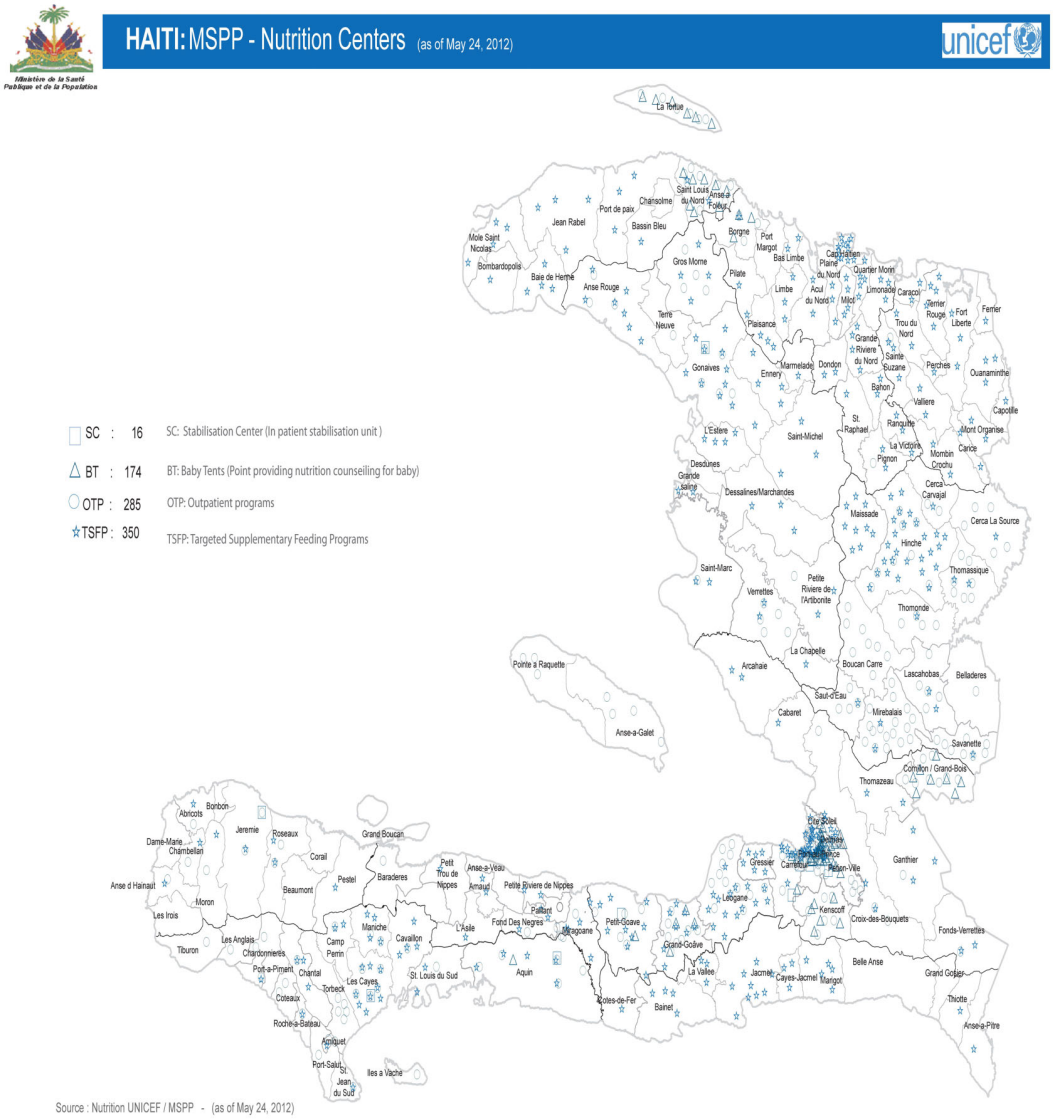


Preventive interventions were provided 6 days a week; curative interventions were provided every day. Nutrition interventions included promotion of optimal breastfeeding practices (early initiation of breastfeeding, exclusive breastfeeding for 6 months, point-of-use food fortification with multiple micronutrient powders to improve complementary foods for breastfed children ages 6–23 months), vitamin A supplementation for children 6–59 months, deworming for children 1–5 years, zinc for the treatment of diarrhea in addition to oral rehydration salt, iron/folic acid supplementation for pregnant and lactating women, ready-to-use supplementary foods, and integrated management of severe acute malnutrition.

## METHODS: COMPARING FINDINGS FROM 2 LARGE SURVEYS

In this study, we sought to assess trends of child undernutrition in Haiti by comparing data from 2005–06 and 2012.

In March 2012, we conducted a nationally representative household survey of child nutritional status using Standardized Monitoring and Assessment of Relief and Transitions (SMART) methodology.^8^^,^^9^ For comparison, we obtained and reanalyzed data from the HDHS 4, conducted by the Haitian Ministry of Public Health and Population (MOPHP) and Macro International between October 2005 and June 2006.^4^ More information about survey design, data collection, and data management is available in the HDHS 4 final report.^4^

Both surveys (HDHS 4 and SMART) applied 2-stage sampling methodologies using the same national household sampling frame, which has been maintained and updated post-earthquake by the Haitian Institute of Statistics. After rural–urban stratification of the data, clusters of at least 25 households were randomly selected from each sampling area. Both surveys collected height and weight data for all children ages 0–59 months living in respondents' households. Interviewers obtained oral consent from parents before collecting children's data.

We calculated the prevalence of stunting (height-for-age z-score <−2 standard deviations [SD]), wasting (weight-for-height z-score <−2 SD), and underweight (weight-for-age z-score <−2 SD) for each survey using World Health Organization 2006 growth standards.^10^ To account for sampling design, we applied probability weights to all analyses, using the SPSS 19.0 Complex Sample Module. We considered differences between the 2 surveys to be statistically significant when 95% confidence intervals did not overlap.

The Haitian MOPHP approved the SMART. ICF International gave permission to use the HDHS 4 data set.

## RESULTS: IMPROVED NUTRITIONAL STATUS

We analyzed growth data for 2,463 (HDHS 4) and 4,727 (SMART) children ages 0–59 months. The table shows sample characteristics, response rates, and findings by survey.

**Table.** Sample Characteristics, Response Rates, and Results, HDHS 2005–06 and SMART 2012

	**HDHS 2005–06**	**SMART 2012**
	**(N = 2,463)**	**(N = 4,727)**
	**% (95% CI)**	**% (95% CI)**
Response rate	99.6	98.0
Age, mean, months^a^	28.2^a^ (27.5–29.0)	26.4^a^ (25.9–27.0)
Sex (female)	51.2	50.4
Rural residence^b^	66.4	57.8
Underweight		
Total	17.7 (15.6–20.1)	10.5 (9.3–11.9)
Urban	12.3 (9.6–15.6)	8.6 (7.4–9.9)
Rural	20.5 (17.7–23.6)	11.7 (10.6–13.0)
Stunted		
Total	28.5 (25.9–31.3)	22.2 (20.2–24.3)
Urban	18.6 (15.3–22.5)	18.4 (16.7–20.1)
Rural	33.6 (30.1–37.2)	25.0 (23.4–26.7)
Wasted		
Total	10.1 (8.2–12.7)	4.3 (3.6–5.2)
Urban	7.5 (5.1–11.1)	4.3 (3.5–5.3)
Rural	11.6 (8.9–15.0)	4.0 (3.3–4.8)

Abbreviations: CI, confidence interval; HDHS, Haitian Demographic and Health Survey; SMART, Standardized Monitoring and Assessment of Relief and Transitions.

a The mean age of children in the SMART sample was significantly lower than that in the HDHS.

b Rural residence: households in villages or non-urbanized areas; urban residence: households in cities and towns.

Between the 2 surveys (2005-06 HDHS and 2012 SMART), the national prevalence of several nutrition-related indicators declined:

- Stunting declined from 28.5% (95% confidence interval [CI] = 25.9, 31.3) to 22.2% (95% CI = 20.2, 24.3)
- Wasting declined from 10.1% (95% CI = 8.2, 12.7) to 4.3% (95% CI = 3.6, 5.2)
- Underweight declined from 17.7% (95% CI = 15.6, 20.1) to 10.5% (95% CI = 9.3, 11.9)

The SMART findings for 2012 are similar to those of the 2012 HDHS,^5^ which was conducted during the same period (see Figure). This similarity supports the robustness of the SMART findings.[Fig f03][Fig f04]

**Figure.**
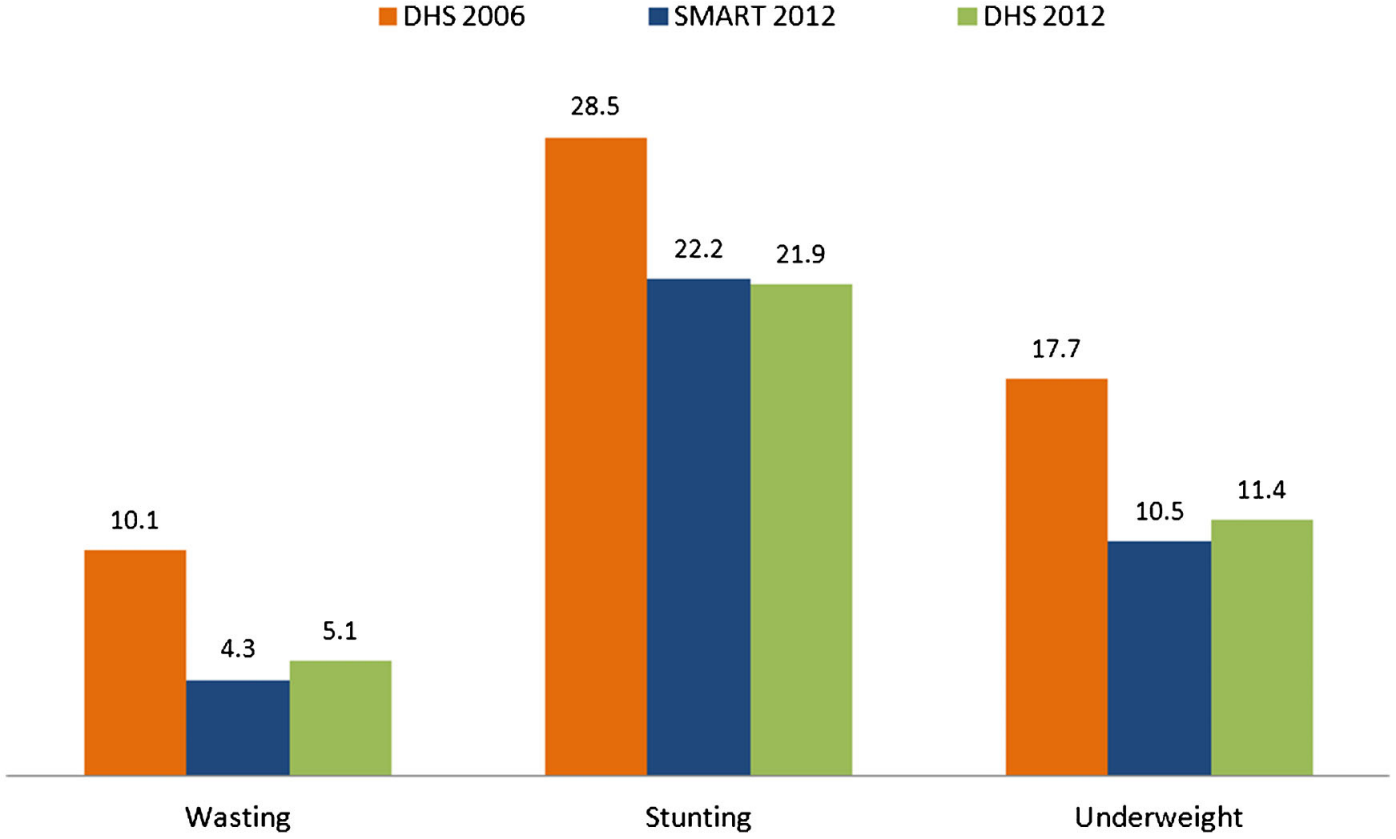
Comparison of Child Nutritional Status (%), 2006 and 2012, Haiti Abbreviations: DHS, Demographic and Health Survey; SMART, Standardized Monitoring and Assessment of Relief and Transitions.

**Figure f03:**
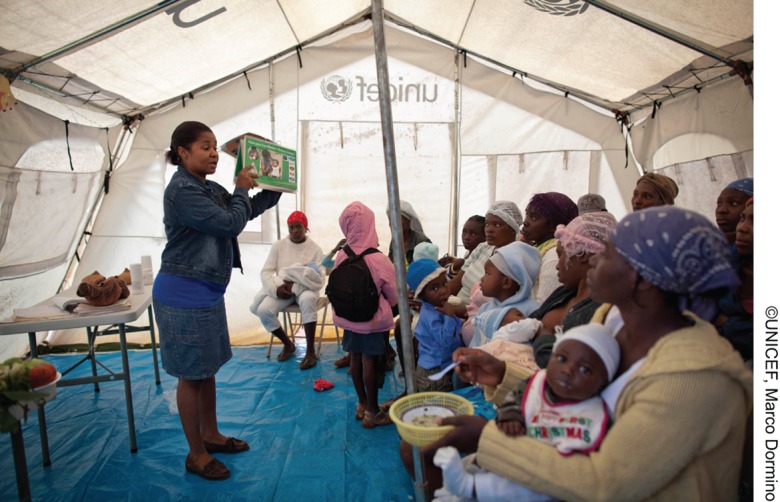
In Haiti, health care workers counseled new mothers on how to provide appropriate complementary foods to their breastfed infants.

**Figure f04:**
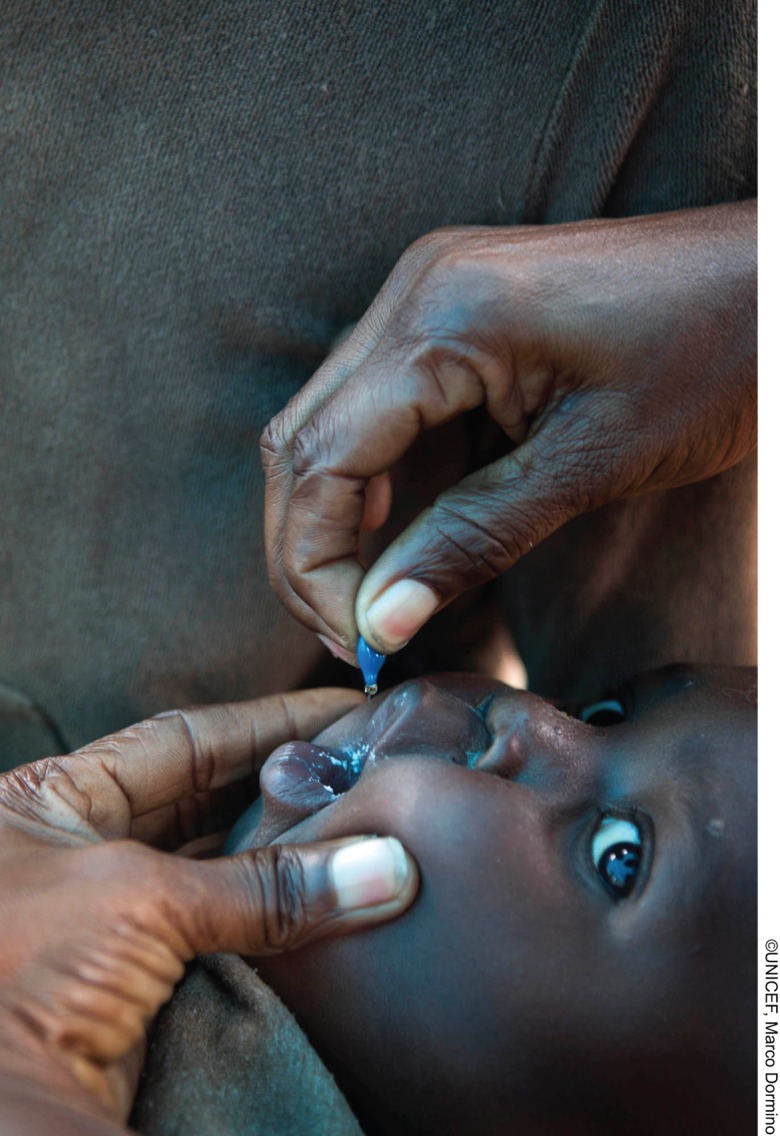
Children between the ages of 6–59 months received vitamin A supplementation.

The rural–urban gap in child undernutrition rates has decreased. For example, in 2005–06, the prevalence of stunting in children under 5, which is associated with adverse long-term child development and economic impacts, in rural areas was 33.6% (95% CI = 30.1, 37.2), while in urban areas it was 18.6% (95% CI = 15.3, 22.5). In 2012, the prevalence of stunting in rural areas was 25% (95% CI = 23.4, 26.7), and in urban areas, it was 18.4% (95% CI = 16.7, 20.1). In other words, in 2005–06 the stunting rate was 15 percentage points higher in rural areas than in urban areas, while in 2012 the rural rate was 6.6 percentage points higher. The 2012 HDHS also found that the gap in rural–urban stunting rates had closed somewhat since the 2005-06 survey. The rural rate in the 2012 HDHS was 24.7%, while the urban rate was 15.6%.^5^

The rural–urban gap in child undernutrition rates has decreased.

## DISCUSSION

The decline between 2005–06 and 2012 in undernutrition among Haitian children 0–59 months old is statistically significant. The decline was more important in rural areas than in urban areas. The reasons for the more rapid decline in rural areas have yet to be elucidated.

At the national level, a number of factors may explain this observed decline in child undernutrition. Between 2006 and 2012, access to health and nutrition services improved,^4^^,^^5^^,^^8^ as evidenced by higher percentages of:

Access to health and nutrition services improved between 2006 and 2012.

- Women attending antenatal care (90.3% in 2012 versus 85% in 2005–06)
- Children immunized against measles (65% in 2012 versus 58% in 2005–06)
- Children with diarrhea treated with oral rehydration salt (52.9% in 2012 versus 40.3% in 2005–06)
- Early initiation of breastfeeding (64.2% in 2012 versus 44.3% in 2005–06)
- Vitamin A supplementation for children ages 6–59 months (44.4% in 2012 versus 28.7% in 2005–06)
- Households using adequately iodized salt (18% in 2012 versus 3.1% in 2005–06)
- Households using improved latrines (26% in 2012 versus 17% in 2005–06)
- Households having access to improved drinking water sources (64.5% in 2012 versus 55.2% in 2005–06)

There also was a slight reduction in the fertility rate (3.5% in 2012 versus 4% in 2005–06).

Furthermore, a few months after the 2010 earthquake, the government developed the National Action Plan for Recovery and Redevelopment,^11^ and, by the end of December 2012, donors had disbursed US$6.4 billion to support Haiti's efforts to restore infrastructure, provide basic social services to the population, and improve the economic situation in the country.^12^ In the months following the earthquake, food was distributed to about 4 million Haitians, and 900,000 received help in the form of cash-for-work or cash transfer to protect overall food consumption,^13^^,^^14^ leading to a quick drop in the percentage of food-insecure households from 52% in February 2010 to 39% in June 2010.^15^

At the same time, major investments were made in integrated health, nutrition, and water, and sanitation and hygiene activities, particularly for the most vulnerable population groups living in camps. For instance, a strategy supported by the United Nations Children's Fund (UNICEF), called “baby tents,” was implemented in earthquake-affected areas, home to more than 65% of the country's population. The strategy protected and improved infant and young child feeding practices. Some 70% of infants less than 6 months of age who participated in the baby tent program were reported to be exclusively breastfed. Furthermore, of those whose mothers initially reported mixed feeding (that is, breast milk plus other foods or liquids), 10% had moved to exclusive breastfeeding before the end of their stay in the program.^16^

UNICEF and the World Food Programme (WFP) also supported implementation of a program for integrated management of acute malnutrition throughout the country (see Map). Results were satisfactory. For example, among the 42,250 severely acutely malnourished children ages 6–59 months enrolled in UNICEF-supported therapeutic feeding programs in 2010, 2011, and 2012, recovery rates were over 75%, death rates were less than 10%, and defaulters were less than 15%.^17^^–^^19^

The nutrition-specific interventions (promotion of optimal breastfeeding practices, micronutrient supplementation, integrated management of acute malnutrition, etc.) and nutrition-sensitive interventions (immunization, diarrhea management, improved access to safe drinking water and sanitation facilities, hygiene promotion, cash transfers, etc.) that were implemented and accelerated after the earthquake are known to have a positive impact on child survival, growth, and development.^20^

Nonetheless, while these interventions reduce child undernutrition, the declines between the 2 surveys cannot be clearly attributed to these interventions alone. Indeed, as has been reported from Brazil,^21^ these findings should be interpreted in light of investments and changes that occurred in different sectors (both within and outside health and nutrition) before and after the earthquake. It is also possible that the declines in undernutrition observed started prior to the earthquake and that the earthquake response accelerated that decline.

A few months after the deadly earthquake, Haiti faced a major cholera outbreak.^3^^,^^22^ One might expect these 2 disasters to worsen children's nutritional status. Alternatively, if the children most likely to die in these disasters were the undernourished, the average nutritional status of the surviving children might appear to have improved over that of the pre-earthquake population. It is not likely, however, that either scenario had a great effect on the nutrition statistics. First, the humanitarian response to the earthquake was swift. Second, an effective treatment protocol for severely malnourished children with cholera was quickly developed and implemented to avert deaths.^23^ The MOPHP indicates a low cholera case fatality rate among children under 5 years—0.7% (638 deaths out of 89,690 cases).^24^ By comparison, among those over age 5, the rate was 1.3% (7,552 deaths out of 577,432 cases).

The earthquake also could have led to rural-to-urban migration in such a way as to reduce the rural–urban gap in nutritional status indicators. However, there are no data available, to our knowledge, that provide evidence of such shifts. In any case, the SMART survey was conducted 2 years after the earthquake; by that time, most of the displaced families had returned home.

There is a need to ascertain the remaining gaps in children's access to services because there is a real potential for pockets of malnutrition in rural as well as peri-urban areas. Our analyses could have been strengthened if the data had allowed comparisons across departments, especially in the relationship between the coverage of interventions and child malnutrition rates. Another potential limitation of our study is that it presents secondary data from just 2 data points, the 2005–06 HDHS and the 2012 SMART.

Pockets of malnutrition may remain in rural as well as peri-urban areas.

While the downward trend in childhood undernutrition in Haiti is encouraging, the overall prevalence of stunting remains high. Sustaining and accelerating the progress made so far will require concerted efforts that target direct and indirect causes of child stunting. These efforts should take into consideration the fact that the distribution of malnutrition is not uniform. Areas and groups still experiencing higher risks or levels of malnutrition need to be identified and assisted.
